# Animal models of chemotherapy-induced cognitive decline in preclinical drug development

**DOI:** 10.1007/s00213-021-05977-7

**Published:** 2021-10-13

**Authors:** Jeena John, Manas Kinra, Jayesh Mudgal, G. L. Viswanatha, K. Nandakumar

**Affiliations:** 1 Department of Pharmacology, Manipal College of Pharmaceutical Sciences, Manipal Academy of Higher Education, Manipal, Karnataka India 576104; 2 Independent Researcher, Kengeri, Bangalore, Karnataka India 560060

**Keywords:** Animal models, Chemobrain, Chemotherapeutic agents, Cognitive impairment

## Abstract

**Rationale:**

Chemotherapy-induced cognitive impairment (CICI), chemobrain, and chemofog are the common terms for mental dysfunction in a cancer patient/survivor under the influence of chemotherapeutics. CICI is manifested as short/long term memory problems and delayed mental processing, which interferes with a person’s day-to-day activities. Understanding CICI mechanisms help in developing therapeutic interventions that may alleviate the disease condition. Animal models facilitate critical evaluation to elucidate the underlying mechanisms and form an integral part of verifying different treatment hypotheses and strategies.

**Objectives:**

A methodical evaluation of scientific literature is required to understand cognitive changes associated with the use of chemotherapeutic agents in different preclinical studies. This review mainly emphasizes animal models developed with various chemotherapeutic agents individually and in combination, with their proposed mechanisms contributing to the cognitive dysfunction. This review also points toward the analysis of chemobrain in healthy animals to understand the mechanism of interventions in absence of tumor and in tumor-bearing animals to mimic human cancer conditions to screen potential drug candidates against chemobrain.

**Results:**

Substantial memory deficit as a result of commonly used chemotherapeutic agents was evidenced in healthy and tumor-bearing animals. Spatial and episodic cognitive impairments, alterations in neurotrophins, oxidative and inflammatory markers, and changes in long-term potentiation were commonly observed changes in different animal models irrespective of the chemotherapeutic agent.

**Conclusion:**

Dyscognition exists as one of the serious side effects of cancer chemotherapy. Due to differing mechanisms of chemotherapeutic agents with differing tendencies to alter behavioral and biochemical parameters, chemotherapy may present a significant risk in resulting memory impairments in healthy as well as tumor-bearing animals.

## Introduction

Cancer is referred as “the modern disease par excellence” or “the quintessential product of modernity.” Although it is considered a modern disease, evidence of its existence in humans and other animals has been found throughout recorded history. Analyzing the timeline of events related to cancer chemotherapy, the beginning of the twentieth century marked the development of cancer models in rodents. Despite the introduction of radical methods like X-rays that focused on the complete eradication of the disease, a crucial turning point for cancer treatment was reached in the 1940s during World War II (WWII).This period initiated chemotherapy, which represented the basis for developing and synthesizing compounds as therapeutic interventions in cancer patients. The discovery of nitrogen mustard during WWII led scientists like Alfred Gilman and Lewis Goodman to examine its therapeutic effects in mice bearing transplanted lymphoid tumors. The post-war drug development program encouraged and introduced different categories of chemotherapeutic agents like folate antagonists and antimitotic agents. In the late 1960s and 1970s, the idea of combination therapy involving multiple drugs with varying mechanisms of action led to further patient improvement and minimal toxicity. Freireich and others designed a program known as VAMP (Vincristine, Adriamycin, Methotrexate and Prednisone) as first in class series of cyclically administered drug treatments that significantly increased the remission rate in children with leukemia. Thereby, studies on combination therapy have eventually paved the way for developing antitumor polychemotherapy, which achieved better therapeutic efficacy over single agents (DeVita and Chu 2008; Galmarini et al. 2012; Ledford 2020).

So, from the initial breakthrough development of chemotherapeutic agents during WWII, there has been significant growth in discoveries concerning the use of novel drugs in cancer treatment. The subsequent developmental stages involving the introduction of targeted therapies, monoclonal antibodies, immune checkpoint inhibitors, cell therapies, and antitumor vaccines in cancer research have improved the survival in cancer patients with sometimes inducing complete tumor remission (Falzone et al. 2018).

With advancements in the global health care system, cancer therapy has significantly impacted survival rates in the past few decades. In the late 1970s and early 1980s, Silberfarb and his colleagues performed a series of studies in cancer patients undergoing chemotherapeutic agents. They emphasized the importance of assessing cognitive functioning as a routine procedure in all cancer patients (Levine et al. 1978). They even demonstrated that cancer patients on the Cognitive Capacity Screening Test had a median score less than non-chemotherapy patients. They further mentioned that patient’s intellectual functioning is often a disregarded part of his/her cancer health care (Silberfarb et al. 1980). So, as more patients survive after cancer treatment, among other different unpleasant side effects, they also experience problems with attention, processing information, and memory that exist for months or even years after the cancer therapy. Thus, cognitive impairment associated with chemotherapy poses one of the biggest challenges for patients trying to get on with day-to-day activities. Apart from CICI, studies have also shown that radiotherapy, hormone replacement therapy, and cancer itself could contribute to cognitive impairment (Shibayama et al. 2014; Hardy et al. 2018; Ganz and Van Dyk 2020).

Chemobrain/chemofog is a general term to explain cognitive impairments with chemotherapy during the treatment or after the treatment experienced by cancer patients/survivors. Cognitive deficits like difficulties with attention, concentration, planning, and working memory were reported in 17 to 75% of breast cancer survivors in a study performed from half a year to 20 years after chemotherapy in these women (Ahles et al. 2012). A study conducted from the time of diagnosis (before chemotherapy) and after completing chemotherapy shows that 45.2% of patients self-reported a deficit in cognitive function compared with 10.4% of healthy people over a similar period (Janelsins et al. 2017). Neuropsychological tests performed in breast cancer survivors found that women exposed to CMF chemotherapy more than 20 years ago reported poorer cognitive performances in verbal memory, processing speed, executive functioning, and psychomotor speed compared to women with no cancer diagnosis (Koppelmans et al. 2012).

Understanding the impact of CICI is one part, and at the same time, striving to unravel its pathophysiology has become another challenge. Unlike other neurodegenerative disorders, CICI does not exhibit characteristics such as personality changes, hallucinations, and motor impairments (stiffness, tremors, falls) (El-Agamy et al. 2019).

It is a transient change that happens as a result of chemotherapy. The onset, duration, and extent of mental dysfunctions caused by CICI vary from person to person. Presently, CICI is theorized as the result of a neuronal injury caused by the alterations in the BBB (blood–brain barrier). This allows the cytotoxic concentrations of the drugs to reach the brain, causing oxidative stress and structural and functional changes in different brain areas (cortex, hippocampus, white and grey matter tracts) that cause perturbance in the executive and memory functions (Holmes 2013). Hippocampus is an important area involved in learning and memory. Altered brain connectivity due to chemotherapy can disrupt the hippocampus’s structure and function, which could directly contribute to impairment in different cognitive domains, particularly long-term and short-term memory. Changes in the prefrontal cortex were also found to be associated with CICI manifesting alterations in memory, attention, and executive functions (Nguyen and Ehrlich 2020; Peukert et al. 2020).

Studies related to chemobrain in humans have limitations because the methodology and small sample sizes of the neuroimaging studies only confirm the existence of cognitive impairment but do not directly describe the mechanism of neuronal injury. Animal models of chemobrain give not only about the critical aspects of pathogenesis but also in assessing the therapeutic efficacy of different agents in attenuating CICI. In a clinical setting, the treatment approach usually involves a blend of chemotherapeutic drugs, making it difficult to assess the individual toxicity or the synergistic effect of different agents. But preclinical conditions aid in evaluating the agents individually and in turn determine their specific mode of actions. Preclinical models also allow us to control variables like age, sex, environment, cancer type, treatment regimens, and comorbid conditions, which are often to be dealt with caution in a clinical setting. Different laboratories can also use distinct strategies to understand CICI, such as using various chemotherapeutic agents, behavioral testing delays, and pharmacological and non-pharmacological therapies through various animal models. However, the development of animal models for CICI is challenging because it is difficult to mimic the human disease, which comprehensively explains the complexities of behavioral, cognitive, and immunological aspects of chemobrain (Winocur et al. 2016; Matsos and Johnston 2019).With this background, this review mainly encompasses the contribution of individual and combination of different chemotherapeutic drugs in inducing post-chemotherapy-related cognitive impairment in healthy and tumor-bearing animals.

## Pathophysiology of chemobrain

Even though different pharmacological mechanisms have been proposed to explain chemobrain, the exact molecular pathways are still unknown. The etiology of chemobrain is multifactorial and defines as the interaction of multiple mechanisms that serve as a logical explanation for its neurotoxic effects rather than a single mechanistic characterization. It is implausible that a single chemotherapeutic agent causes cognitive dysfunction because the patient receives a combination of different drugs, mainly depending on the molecular mechanisms of drugs’ on-target and off-target effects and synergistic consequences of drugs. This section will discuss three main contributing factors responsible for inducing CICI (Wang et al. 2015).

### Inflammation

Chronic inflammation is a common characteristic responsible for cognitive dysfunction in most neurodegenerative disorders. Chemotherapeutic agents upregulate pro-inflammatory cytokines in the hippocampus, frontal cortex, and corpus callosum, which regulate different cognitive functions. In cancer patients, multiple mechanisms increase the cytokine levels, and even the administration of chemotherapy agents that mainly focus on cancer cell death can also alter cytokine levels (Cleeland et al. 2003). Higher levels of inflammatory cytokines are directly correlated with impaired cognitive function. However, most chemotherapeutic agents like doxorubicin and methotrexate, when administered systemically, do not cross the BBB. Nonetheless, it is said that there is a communication between the peripheral cytokines and central cytokines because many studies have shown an elevation in cytokine levels such as TNF-α (tumor necrosis factor-α) and IL-6, IL-8, IL-10, (interleukins), and MCP-1 (monocyte chemoattractant protein-1) in cancer patients and were prominent in people with dyscognition. This may further instigate changes in the neurotransmitter levels, oxidative parameters, neuronal growth factors, and neuronal integrity directly correlated with the neuronal and cognitive functions (Wang et al. 2015). So, the indirect route of induction of chemobrain via peripheral cytokines is not adequately explored, and further studies are required to provide a detailed explanation on this area. Chemotherapeutic agents like 5-fluorouracil, cyclophosphamide, carmustine, cisplatin, cytarabine can cross BBB and cause direct neurotoxicity. In addition to this, different clinical studies have shown cytokine progression in other forms of cancer even before chemotherapy treatment. So, more focus should be laid on whether the cognitive changes due to inflammatory cytokines are attributed only by chemotherapeutic agents or due to the progression of cancer itself (Cheung et al. 2015).

### Impaired neurogenesis

In the brain, adult neurogenesis takes place in two critical areas:

- Subgranular zone of the dentate gyrus of the hippocampus
- Subventricular zone lining the lateral ventricles and striatum

Reduced neurogenesis also plays an essential role in aging and neurodegeneration. As age increases, neurogenesis declines because it acts as a buffer for maintaining the functions of neurons. Chemotherapeutic agents have been found to accelerate the physiological aging process. Different studies have established a direct correlation between reduced neurogenesis and CICI (Seigers et al. 2008; ElBeltagy et al. 2010; Nokia et al. 2012). A wide range of chemotherapeutic agents like doxorubicin, cyclophosphamide, carmustine, cisplatin, and 5-fluorouracil have been reported to interfere with hippocampal neurogenesis causing altered levels of neurogenesis markers like Ki-67, BrdU in different preclinical studies (Ahles and Saykin 2007; Mounier et al. 2020). Many chemotherapeutic agents like paclitaxel, docetaxel, vincristine, and vinblastine target extensive microtubule-based network, which is required for proper axonal transport, communication, and functioning of the neurons. Since most chemotherapy agents are developed to stop cell division, they may also block neurogenesis and gliogenesis. Moreover, the patients with colon cancer and brain tumor have shown reduced hippocampal volume and neurogenesis followed by chemotherapy (Schneiderman 2004; Penzes et al. 2011). Brain-derived neurotrophic factor (BDNF), a neurotrophin, is responsible for maintaining synaptic plasticity in the brain. Chronic treatment with chemotherapeutic agents causes a deficit in central BDNF levels, affecting neuronal functions (NG et al. 2015). Approaches to resume the BDNF levels in the brain by certain drugs such as antidepressants (Fluoxetine) have been discussed and provide their scope of implication as a therapeutic strategy for CICI (Das et al. 2020).

### Oxidative stress

An imbalance between reactive oxygen species and antioxidants is known to cause oxidative stress. Normal redox state plays a vital role in maintaining brain functions. A study conducted in the blood serum samples collected from patients with Hodgkin’s lymphoma and multiple myeloma subjected to chemotherapy has decreased glutathione peroxidase, catalase, and superoxide dismutase which constitute an antioxidant defense system (Santos et al. 2016). Disturbances in antioxidant mechanisms by chemotherapeutic agents result in the generation of free radicals in the hippocampus and frontal cortex involved in learning and memory. Apart from these regions, the accumulation of reactive oxygen species (ROS) occurs in blood serum and neural tissue homogenates. Increased lipid peroxidation and decreased levels of reduced glutathione, catalase, and superoxide dismutase cause mitochondrial derangement, which finally disrupts cellular metabolic processes (Gaman et al. 2016). Most of the chemotherapeutic agents like doxorubicin, cyclophosphamide, carboplatin, cytarabine, and methotrexate have been reported to produce ROS in both periphery as well as in central nervous system (CNS) and results in cognitive impairment (Barton and Loprinzi 2002; Mounier et al. 2020). Also, chemotherapy-induced mitochondrial abnormalities as a result of oxidative stress can affect neuronal and cognitive functions. Loss of neuronal and synaptic plasticity takes place due to calcium exocytosis and ionic imbalance in the mitochondria (Dietrich et al. 2006).

Chemotherapeutics may affect the white and gray matter integrity due to decreased cortical spines and dendrites and alter the cortex-based performances (Nguyen and Ehrlich 2020). Interestingly, loss of dendritic integrity is reported in different neurodegenerative conditions (Dumanis et al. 2009). Apart from neurodegeneration, neuronal signaling and long-term potentiation (LTP) may also get distorted due to chemotherapy. Evidence support that CICI may be implicated to interfere with LTP (Lee 2006). The neurological process of learning and memory depends upon synaptic plasticity, and larger synaptic efficiency is involved in the form of LTP. So, the inappropriate loss of synaptic activity or the abnormalities in the induction and maintenance of LTP may result in cognitive impairment (Fahim et al. 2019).

**Fig. 1:**
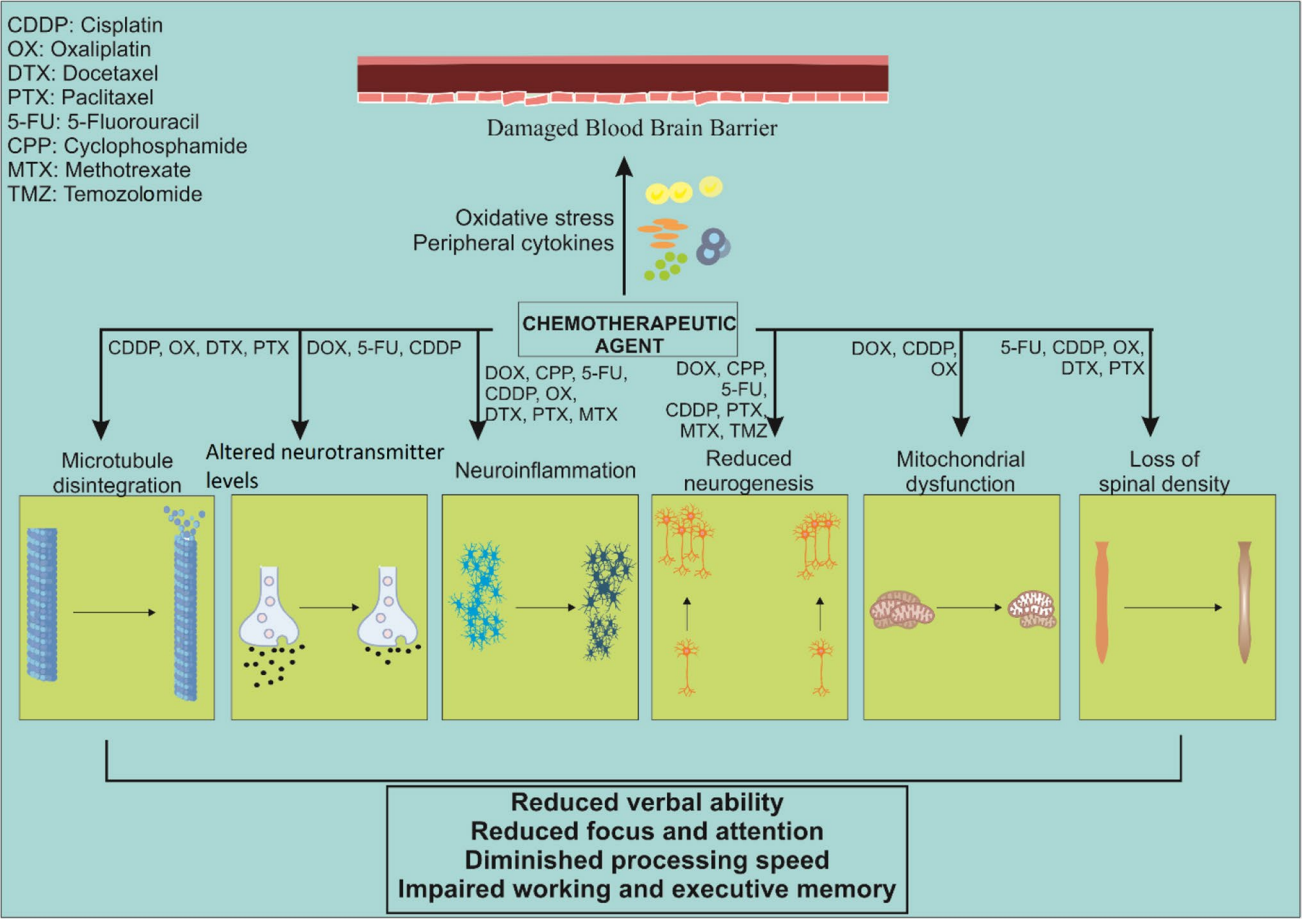
Possible cellular mechanisms of chemobrain and the resulting cognitive deficits

Even though the chemotherapy-induced metabolic alterations are acute, their consequence manifested as cognitive impairment is chronic. This introduces epigenetics, which has already been proven to regulate gene expression in various brain disorders. Cognitive dysfunction following cyclophosphamide, methotrexate, and 5-fluorouracil-based chemotherapy was related to increased histone H3 acetylation and reduced histone deacetylase activity hippocampus in an animal model. It can be a likely mechanism of chromatin remodeling in resulting long-term cognitive deficits after chemotherapy due to a transitory cytokine change (Briones and Woods 2011; Verma et al. 2017). Most chemotherapeutic agents have been linked to affecting neurons by multiple mechanisms, and the subsequent effect of CICI may be attributed to the combination of different neurotoxic insults (Fig. 1).

### Animal models of chemobrain

**Fig. 2:**
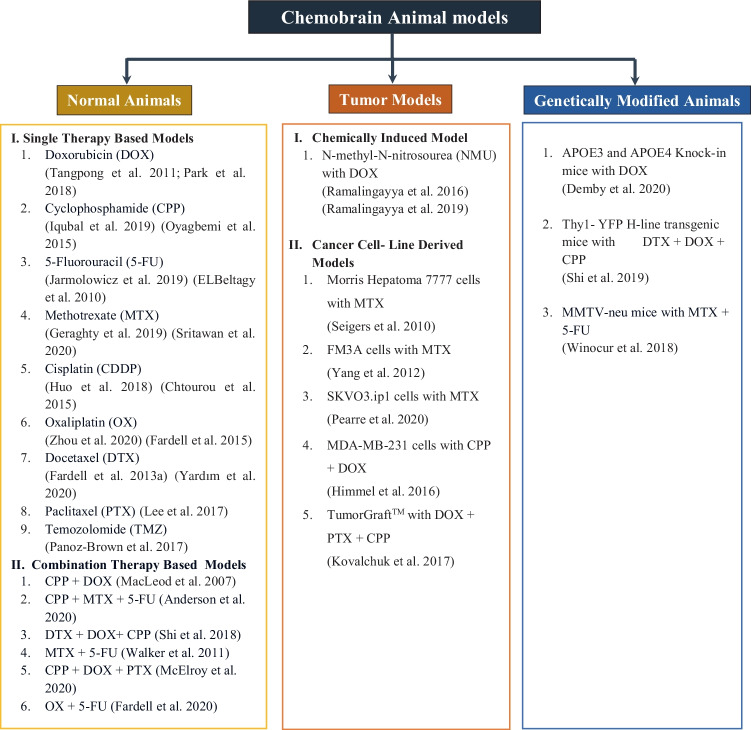
Classification of animal models of chemobrain

An ideal animal model of chemobrain should reflect the different mechanisms of actions exerted by chemotherapeutic agents. Various animal models utilizing a broad range of chemotherapy agents have been developed based on distinct pathophysiological mechanisms (Fig. 2).

### Inclusion and exclusion criteria

The literature search was limited to research and review articles, excluding references in languages other than English. The subjects included in the study were rodents (mice, rats). Only those studies that describe the behavioral and biochemical tests for establishing the effect of chemotherapy on cognitive function in animals were included in preparing the manuscript. Studies that report the role of surgery, radiation, and hormone replacement therapy on cognitive function were excluded from the data analysis.

### Induction of chemobrain in healthy animals

Single therapy chemobrain models provide a precise mechanism of the respective chemotherapeutic agent responsible for inducing cognitive deficits. In addition, the effect of commonly used chemotherapeutic agents in healthy animals may predict the extent of dyscognition caused by each agent in the absence of a tumor. This sort of analysis is necessary to develop treatment strategies that can improve cancer survivors’ quality of life.

#### Doxorubicin

Doxorubicin (DOX) is a ruling chemotherapeutic agent used in several carcinomas like ovarian, prostate, thyroid, and Hodgkin’s disease. It exerts a robust anti-cancer effect and damages healthy tissues throughout the body resulting in cardiotoxicity, hepatotoxicity, and nephrotoxicity (Carvalho et al. 2009). The structure of DOX-containing quinone makes it susceptible to redox cycling and contributes to excessive ROS production resulting in cellular biomolecule modifications (Mizutani et al. 2005). Even though DOX is impermeable to the BBB, the peripheral cytokines being generated due to its cell toxicity result in an indirect neurotoxic effect (El-Agamy et al. 2019).

DOX is a member of the anthracycline family, and its cytotoxicity is thought to be based on the following paradigms (Keizer et al. 1990; Mizutani et al. 2005):

1. Intercalation into DNA thus interfering in cancer growth and division
2. Topoisomerase II inhibition thereby interfering in DNA unwinding steps
3. Generation of excess amount of ROS which exerts anti-tumor activity and may also result in other off-target effects

In vitro studies show that DOX exerts its toxicity in tumor cells by inducing p53 dependent apoptosis (Brantley-Finley et al. 2003), whereas its toxicity in non-cancerous tissue is p53 independent (Wang et al. 2004). A recent meta-analysis study to determine the cognitive performance among the DOX-treated women with breast cancer reported significant cognitive impairment, including processing speed, language, memory, and executive functioning compared with healthy controls (Eide and Feng 2020).

DOX-induced chemobrain animal models have reported that DOX mechanistically induces dysregulation of cytokine and neurotransmitter levels consequently inducing behavioral alternations (Table 1).

**Table 1:** Different animal models of doxorubicin inducing cognitive dysfunction

Species	Dose & route of administration	Comments	Reference
Male Sprague–Dawley rats (200–250 g)	2 mg/kg/week, *i.p* for 4 weeks	• Enhanced NF-κb (p65), COX-II/TNF-α, GFAP • Inhibition of LTP in hippocampus and dentate gyrus	(Ali et al. 2020)
Female Wistar rats (12 weeks old)	2.5 mg/kg, *i.p* for once in 5 days for 50 days	• Increased levels of TNF-α in hippocampus and frontal regions • Impaired episodic memory in NORT	(Ramalingayya et al. 2017)
Male B6C3 mice (8 weeks old)	Single injection 20 mg/kg, *i.p*	• Increased caspase-3, TUNEL-positive cells, TNF-α, p53, Bax levels • Decline in brain mitochondrial respiration	(Tangpong et al. 2011)
Male Wistar rats (6 weeks old)	3.5 mg/kg/week, *i.p* for 8 weeks	• Elevated levels of TNF-α, iNOS and COX-2	(Leung et al. 2020)
Male Sprague–Dawley rats (6 weeks old)	2 mg/kg/week, *i.p* for 4 weeks	• Impaired spatial memory in MWM task	(Tong et al. 2020)
Male Wistar rats (2–3 months old)	Single escalating doses 0.8,2, 8 mg/kg, *i.p*	• Impaired long-term aversive learning • Impaired innate exploratory behavior	(Liedke et al. 2009)

A systemic administration of DOX (2 mg/kg/week, *i.p*) for 4 weeks in Sprague–Dawley rats triggered oxidative stress evidenced by increased lipid peroxidation and reduced glutathione levels. Treatment with DOX also resulted in enhanced NF-κb (p65) (nuclear factor kappa-light-chain-enhancer of activated B cells) nuclear translocation factor, pro-inflammatory cytokine (COX-II/TNF-α) levels, and glial fibrillary acid protein (GFAP), which finally resulted in neuroinflammation. CICI is mainly mediated through oxidative stress by TNF-α elevation, which is reported to inhibit LTP in the hippocampus and dentate gyrus (Ali et al. 2020). Tangpong et al. showed an enhanced level of caspase-3, TNF-α, and TUNEL-positive cells after 72 h of treatment with a single injection of Adriamycin (20 mg/kg, *i.p*) (Tangpong et al. 2011). TNF-α levels were significantly elevated in hippocampal and frontal regions of the female Wistar rats when treated with DOX (2.5 mg/kg, *i.p*) for once in 5 days for 50 days (Ramalingayya et al. 2017). Leung et al. reported that male Wistar rats, when treated with DOX (3.5 mg/kg/week, *i.p*) for 8 weeks, showed elevated levels of stress-related inflammatory proteins TNF-α, iNOS (inducible nitric oxide synthase) and COX-2 (cyclooxygenase-2) (Leung et al. 2020).

Hippocampus is the primary site of neurogenesis and is involved in neuronal plasticity. To assess the extent of alterations in the neuroplasticity and neurogenesis in the dentate gyrus, DOX was administered in Wistar rats (2 mg/Kg/week, *i.p*) for 4 weeks and resulted in cognitive deficits accompanied by a decrease in the levels of BDNF and TrKB (tropomyosin-related kinase-B) (Park et al. 2018). Glutamate is the primary excitatory neurotransmitter in the frontal cortex and hippocampus, which predominantly mediates cognitive function. Single acute injection of DOX (25 mg/kg/, *i.p*) in mice showed a decreased clearance of glutamate in the frontal cortex and dentate gyrus assessed 24-h post-injection by employing novel glutamate-selective microelectrode arrays (Thomas et al. 2017).

Mitochondria play a vital role in the survival of neurons. So, disruption/dysregulation of mitochondrial respiration can lead to apoptotic cell death resulting in excess production of superoxide, hydrogen peroxide, and reactive oxygen species (ROS). It may lead to the opening of mitochondrial permeability transition pores (PTP), causing uncontrolled leakage of cellular constituents and resulting in mitochondrial damage (Javadov and Kuznetsov 2013). Studies in B6C3 mice treated with a single intraperitoneal injection of Adriamycin (20 mg/kg) led to the decline in brain mitochondrial respiration along with the rise in pro-apoptotic proteins p53 and Bax. It also showed the translocation of p53 and its interaction with anti-apoptotic protein Bcl-XL accompanied by the release of cytochrome-c and initiating cell death with the generation of O2^−^ (Tangpong et al. 2006). Seven weekly subcutaneous injections of DOX (2 mg/kg) in male Wistar rats enhanced brain mitochondrial sensitivity to Ca^2+^-induced PTP opening culminating in neuronal degeneration (Cardoso et al. 2008). Manganese superoxide dismutase, a mitochondrial localized antioxidant enzyme, was nitrated in mice treated with a single injection of DOX (20 mg/kg, *i.p*) (Tangpong et al. 2007).

DOX has also been reported to induce a wide range of functional impairments across a broad range of cognitive domains. Ramalingayya et al. reported episodic memory deficits in female Wistar rats on chronic administration of ten cycles of DOX (2.5 mg/Kg, *i.p*) in the Novel Object Recognition Task (NORT) evidenced by decreased exploration time and discrimination index with the novel object (Ramalingayya et al. 2017). DOX (2 mg/kg/week, *i.p*) treated in athymic nude rats for 4 weeks showed impaired performance (reduced freezing behavior) in the contextual fear conditioning task (Christie et al. 2012). The role of DOX in impairing long-term aversive learning was reported in the inhibitory avoidance conditioning task in male Wistar rats after administering single escalating doses of DOX (0.8,2, 8 mg/kg, *i.p*). DOX impaired the animal’s learning capacity before training, which was measured on day 1 and day 7. In the same study, DOX decreased the innate exploratory behavior in rats determined by the number of rearings performed in the Open Field task (Liedke et al. 2009). Spatial memory assessed by Morris Water Maze (MWM) showed that DOX treatment (2 mg/kg/week, *i.p*) for 4 weeks in male Sprague–Dawley rats significantly increased the escape latency when compared to the control group (Tong et al. 2020). It has also been reported that C57BL/6 J mice when subjected to DOX (4 mg/kg/week, *i.p*) for 3 weeks preserved learning and memory assessed during fear conditioning task and NORT which showed no significant difference in the percentage of freezing time and novel object preference (exploration time), respectively. This shows the possible resistance of C57BL/6 J mice to some aspects of CICI as observed in some cancer patients (Fremouw et al. 2012).

#### Cyclophosphamide

Cyclophosphamide (CPP) is one of the frequently used chemotherapeutic agents mainly indicated to treat different types of malignant lymphomas like non-Hodgkin and Hodgkin lymphomas, Burkitt lymphoma, lymphocytic lymphoma, and multiple myeloma, which is either used alone or in combination with other chemotherapeutic agents. Apart from this, CPP is used for breast cancer, retinoblastoma, and ovarian adenocarcinomas. In addition to its anti-neoplastic effects, CPP also has immunosuppressant actions to prevent transplant rejections in autoimmune disorders like multiple sclerosis (Ogino and  Tadi 2021).

Unlike DOX, CPP crosses the BBB, and the dyscognition induced owing to CPP treatment may be due to the indirect tissue toxicity, oxidative damage, and a direct injury to neurons. It alkylates DNA and prevents replication of the genome. CPP also blocks the S phase of the cell cycle and aids in apoptosis (Ueno et al. 2006). CPP metabolite acrolein/phosphoramide mustard induces oxidative stress, damaging the BBB, making it susceptible to neurotoxic molecules’ entry into the brain (Subramaniam et al. 1994).

Different animal models have reported that CPP induces dysregulation of cytokine and neurotransmitter levels, consequently inducing behavioral alterations (Table 2).

**Table 2:** Different animal models of cyclophosphamide inducing cognitive dysfunction

Species	Dose & route of administration	Comments	Reference
Male Swiss mice (35–40 g)	Single injection 200 mg/kg, *i.p*	• Enhanced NF-κb, TNF-α, IL-6, and IL-1β in frontal cortex and hippocampus • Reduced retention transfer in step-down latency test	(Iqubal et al. 2019)
Male Wistar rats (100–145 g)	Single injection 100 mg/kg, *i.p*	• Increased levels of MDA, nitrites, and hydrogen peroxide • Reduced levels of catalase, superoxide dismutase, and glutathione S transferase	(Oyagbemi et al. 2015)
Male C57BL/6 mice (6–8 weeks old)	200 mg/kg, *i.p* for 4 weeks	• Decline in spatial memory in reward-based spatial alternation paradigm	(Janelsins et al. 2016)
Male Sprague–Dawley rats (6–8 weeks old)	25,50 mg/kg/week, *i.p* for 4 weeks	• Suppression of hippocampal neurogenesis • Impairment of spatial memory in MWM	(Wu et al. 2017)
Male Swiss Albino mice (6–8 weeks old)	Single injection 75 mg/kg, *i.p*	• Increased levels of MDA, hydroperoxides and conjugated dienes	(Bhatia et al. 2006)
Male ICR mice(8–10 weeks old)	Single injection 40 mg/kg, *i.p*	• Decreased levels of Ki-67 and DCX in hippocampus	(Yang et al. 2010)

Neuroinflammatory properties of CPP which were assessed by administering CPP (200 mg/kg, *i.p*) for once (on the 7th day of the treatment protocol) in male Swiss mice showed elevated levels of inflammatory cytokines such as TNF-α, IL-6, and IL-1β and reduced the level of anti-inflammatory cytokine IL-10 with enhanced expression of NF-κB in the frontal cortex and hippocampus. CPP administration caused significant damage to neuronal architecture and resulted in neuronal degeneration revealed by the histological findings in the hippocampus and frontal cortex. Researchers also reported that CPP-treated group significantly decreased the retention transfer latency in the step-down latency test (Iqubal et al. 2019). A single injection of CPP (100 mg/kg, *i.p*) administration in male Wistar rats showed a significant elevation in the levels of cerebral and cerebellar malondialdehyde (MDA) contents, nitrites, and hydrogen peroxide generation. Along with this, catalase, superoxide dismutase, and glutathione S transferase levels were reduced in the CPP-treated brain (Oyagbemi et al. 2015). Acute administration of CPP (75 mg/kg, *i.p*) increased the levels of MDA, hydroperoxides, and conjugated dienes in the mouse (Swiss Albino) brain (Bhatia et al. 2006).

C57BL/6 mice treated with CPP (200 mg/kg, *i.v*) for 4 weeks at weekly intervals using a reward-based delayed spatial alternation paradigm with delay values of 1.5,3, 6.1, 12.4, and 25 s over 80 sessions showed a decline in spatial memory at the most extended delay as the mice ages (Janelsins et al. 2016). For 4 weeks, male Sprague–Dawley rats treated with CPP (25,50 mg/kg/week, i.p) found a long-lasting cognitive impairment with suppression of hippocampal neurogenesis confirmed by decreased doublecortin (DCX)-positive cells in the hippocampal dentate gyrus. Spatial memory assessed using MWM revealed that CPP-treated group took a long time to reach the escape platform compared to the control group (Wu et al. 2017). Mice treated with CPP (100 mg/kg, *i.p*) for every 2 alternate days (4 scheduled doses) showed cognitive deficits when tested in different behavioral paradigms such as Y maze (ability to recognize places already explored), and Norwegian Tenecteplase Stroke trial test (hippocampal-dependent memory) (Alhowail et al. 2019). In another study to understand the behavioral alterations in learning and memory induced by chemotherapy, CPP (40 mg/kg, *i.p*) was administered in mice and showed cognitive deficits in passive avoidance and object recognition task 12 h after injection. They have also reported that CPP subtly decreased the levels of immunohistochemical markers of neurogenesis such as Ki-67 and DCX in the hippocampus, indicating that CPP induced hippocampus-dependent memory dysfunction (Yang et al. 2010).

#### 5-Fluorouracil

5-Fluorouracil (5-FU) is an antimetabolite commonly used in the treatment of colorectal and breast cancers. It blocks the thymidylate synthase enzyme responsible for converting deoxyuridine monophosphate (dUMP) to thymidine monophosphate (dTMP) for the synthesis of the DNA base, thymidine. It mainly acts by the misincorporation of fluoronucleotides in RNA and DNA, thereby interfering with DNA replication and functioning (Longley et al. 2003). 5-FU crosses BBB by simple diffusion. Cognitive deficits have been reported when used as a high-dose single agent and combined with other agents (Wigmore et al. 2010). Different animal studies have reported that 5-FU induces cognitive impairment, causing dysregulation of cytokine and neurotransmitter levels and subsequent behavioral alterations (Table 3).

**Table 3:** Different animal models of 5-fluorouracil inducing cognitive dysfunction

Species	Dose & route of administration	Comments	Reference
Male Wistar rats (5 months old)	25 mg/kg, *i.v*	• Impaired attentional shifting • Reduced dopamine release	(Jarmolowicz et al. 2019)
Male Lister hooded rats (200–250 g)	Five injections 25 mg/kg, *i.v* for 2 weeks	• Induction of spatial memory deficits in OLT • Reduced levels of DCX and BDNF in hippocampus	(Mustafa et al. 2008)
Male Lister hooded rats (150–170 g)	Five injections 25 mg/kg, *i.v* for 2 weeks	• Decrease in freezing time in context-dependent conditional-response test • Reduced levels of Ki67 in subgranular zone of dentate gyrus	(ELBeltagy et al. 2010)
Male CBA mice (6–8 weeks old)	40 mg/kg, *i.p* of 3 injections over 6 months	• Induction of apoptosis in subgranular zone of dentate gyrus • Loss of myelin integrity	(Han et al. 2008)
Male C57B16/J mice (6 months old)	60 mg/kg, *i.p* thrice over 3 weeks	• Reduction in spine density • Elevation in IL-17, IL-1β, and GMCSF in hippocampus	(Groves et al. 2017)

Studies show that alterations in dopaminergic signaling and conduction which is necessary for motivation and learning also contribute to chemotherapy-induced cognitive impairment. Male Wistar rats were treated with 5-FU (25 mg/kg, *i.v*) to understand the effects of chemotherapy in attentional shifting, which is an integral part of executive function. The results showed that 5-FU impaired attentional shifting. Behavioral outcomes were also compared with the extent of dopamine release measured using fast-scan cyclic voltammetry, which showed decreased dopamine release persisted for a week after the second injection (Jarmolowicz et al. 2019).

5-FU has been reported to alter cell proliferation and neurogenesis in the dentate gyrus, resulting in a decline in cognitive performance. This was supported by treating five injections of 5-FU (20 mg/kg, *i.v*) for 12 days in male Lister hooded rats and showed a decrease in DCX and BDNF levels in the hippocampus. Induction of spatial memory deficits was demonstrated by the inability to discriminate the objects placed in the novel and familiar locations in object location task (OLT) (Mustafa et al. 2008). Male Lister hooded rats treated with five injections of 5-FU (25 mg/kg, *i.v*) for 2 weeks showed a decrease in freezing time in a context-dependent conditional emotional response test compared with the control group. It confirms the involvement of the hippocampus in the memory impairment produced by 5-FU. They have also reported a decreased level of cell proliferative marker, Ki67, in the subgranular zone of the dentate gyrus (ELBeltagy et al. 2010).

Using autoshaping operant procedure and progressive ratio procedure, mice treated with 5-FU (75 mg/kg, *i.p*) were trained to respond for a reward in the presence of audible tone on day one as a measure of learning and the same performed on day 22 as a measure of memory retention in the absence of tone. The results showed that 5-FU significantly increased the time required to retrieve previously learned behavior (Foley et al. 2008).

A single administration of 5-FU (75 mg/kg, *i.p*) in male hooded Wistar rats showed significant impairments in the episodic memory tested in NORT. Spatial memory deficits were also assessed in the same study using MWM and showed increased escape latency (Fardell et al. 2012). Treatment of 5-FU (40 mg/kg, *i.p*) of 3 injections over 6 months in mice caused significant induction of apoptosis in the subventricular zone of the dentate gyrus in the hippocampus persisted for 14 days. The same study also reported that 5-FU caused delayed changes in OLIG2 (oligodendrocyte transcription factor-2) expression and resulted in the loss of myelin integrity (Han et al. 2008). C57B16/J mice treated with 5-FU (60 mg/kg, *i.p*) thrice over 3 weeks showed disadvantageous effects of 5-FU on neurons by reducing spine density and increasing inflammatory cytokines affecting hippocampal learning and memory. This was confirmed by the decrease in the density of mushroom spines in the dentate gyrus and the overall reduction in spine density in CA1 apical pyramidal dendrites and CA3 apical neurons. The levels of interleukins like IL-17, IL-1β, and GMCSF (granulocyte–macrophage colony-stimulating factor) were also increased in the hippocampus (Groves et al. 2017).

#### Methotrexate

Methotrexate (MTX) is an antifolate inhibitor of dihydrofolate reductase (DHFR) employed in the treatment of children with acute lymphoblastic anemia (ALL), non-Hodgkin’s lymphoma, and osteosarcoma. DHFR is involved in converting dihydrofolate (DHF) to tetrahydrofolate (THF), which is needed for purine biosynthesis and acts as the precursor for dTMP synthesis. So, MTX acts as a competitive inhibitor of DHF and interferes in purine and pyrimidine biosynthesis, resulting in cytotoxicity (Breedveld et al. 2004).

MTX administration caused long-term deficits in attention, working memory, and executive function for around 50–70% of pediatric ALL survivors (Hearps et al. 2016; Van Der Plas et al. 2015). This was supported in a preclinical setup that reported single MTX administration (25 mg/kg, *i.p*) caused long-term cognitive dysfunction in rats (Fardell et al. 2010). The likelihood of MTX crossing the BBB is low (Angelov et al. 2009), but a high dose of MTX administration can reach the required brain concentration (Comandone et al. 2005). Preclinical studies have documented the potential effects of MTX in inducing cognitive impairment and associated biochemical and behavioral changes (Table 4).

**Table 4:** Different animal models of Methotrexate inducing cognitive dysfunction

Species	Dose & route of administration	Comments	Reference
Male Wistar rats (200–220 g)	0.2 mg/kg/day, *s.c* for 7 days	• Increased peroxidation and reduction in GSH/GSSG ratio	(Rajamani et al. 2006)
Male C57BL/6 & CD1 mice	100 mg/kg, *i.p*	• Depletion of OPCs in white matter • Disruption of neuroplasticity • Chronic microglial activation	(Gibson et al. 2019)
Male Lister hooded rats (150–200 g)	2 doses 75 mg/kg, *i.v*	• Impairment of hippocampal neurogenesis • Reduced levels of Ki67, DCX, BrdU • Impaired spatial memory in OLT	(Lyons et al. 2010)
Male Sprague–Dawley rats (180–200 g)	Single injection 100 mg/kg, *i.p*	• Increased endoplasmic stress and apoptosis • Spatial memory impairment in MWM	(Lv et al. 2020)
Female C57B16/J mice (1 month old)	Single injection 200 mg/kg, *i.p*	• Folate depletion • Elevation in CSF tau and reduced hippocampal cell proliferation	(Elens et al. 2019)
Male Sprague–Dawley rats (4–5 weeks old)	Two doses 75 mg/kg/day, *i.v*	• Spatial and episodic memory deficits in OLT and NORT respectively	(Sritawan et al. 2020)

Administration of MTX (0.2 mg/kg/day, *s.c*) in male Wistar rats for 7 days showed increased peroxidation level and reduction in GSH/GSSG ratio in different regions of the brain (cortex, cerebellum, midbrain, hippocampus) (Rajamani et al. 2006). Similarly, increased oxidative stress and impaired executive function were reported in children receiving MTX therapy for ALL (Caron et al. 2009).

A C57BL/6 and CD1 mouse model of MTX (100 mg/kg, *i.p*) reveals that oligodendrocyte precursor cells (OPCs) were depleted in the white matter, which leads to impairments in myelination and disruption in neuroplasticity. MTX induced chronic microglial activation and disruption of glial cell homeostasis. The study has also reported that MTX-treated mice did not differentiate between the novel and familiar objects in NORT (Gibson et al. 2019). In an extension of this study, researchers found that MTX caused a reduction in microglia-dependent BDNF expression and impaired cognitive-behavioral performance (Geraghty et al. 2019).

Intrathecal administration of MTX (10 mg/kg) into C57BL/6 mice (21 days old) reported impaired hippocampal-dependent cognition assessed using MWM test showed the decreased ability of mice to find the hidden platform (Alexander et al. 2018). Male Sprague–Dawley rats treated with MTX (75 mg/kg/day, *i.v*) on the 7th and 14th days of 36 days treatment period failed to differentiate the novel and familiar object locations in the OLT. In the NORT, rats could not discern between the novel and familiar objects in the choice trial confirming the CICI (Sritawan et al. 2020).

MTX impaired hippocampal neurogenesis by reducing different immunohistochemical markers like Ki67, BrdU (bromodeoxyuridine), and DCX. This was supported by administering two doses of MTX (75 mg/kg, *i.v*) in male Lister hooded rats, given a week apart. Researchers found that MTX induced spatial memory deficits by showing no preference for either the novel or familiar location in the choice trial of OLT. MTX also decreased the Ki67-positive cells in the subgranular zone of the dentate gyrus (Lyons et al. 2010).

Male Sprague–Dawley rats were treated with a single injection of MTX (100 mg/kg, *i.p*) and found to increase the endoplasmic stress and induced apoptosis confirmed by the presence of a more significant number of TUNEL-positive cells. In the hippocampus, MTX increased the expression of CHOP (CCAAT/enhancer-binding protein (C/EBP)-homologous protein) and cleaved caspase 12 and decreased the expression of DCX- and Ki67-positive neurons in the dentate gyrus. Spatial memory impairments were assessed in the same study using MWM, where the rats with MTX showed a significant decrease in the time spent in the target quadrant compared to the vehicle-treated group (Lv et al. 2020). Five-week-old Sprague–Dawley rats when treated with MTX (75 mg/kg, *i.v*) induced changes in hippocampal neurogenesis and behavioral parameters shown by memory deficits in NORT and OLT shown by decrease in preference index as well as decline in neurogenesis markers like Ki67-, BrdU-, and DCX-positive cells (Naewla et al. 2019). Acute (25 mg/kg, *i.p*) and chronic (1 mg/kg, *i.p*, twice weekly, four doses) administration of MTX in long Evans rats produced spatial memory deficits in OLT and NORT and showed a significant reduction in the serum and CSF folate concentrations (Li et al. 2010). Folate is necessary for normal brain development and is involved in the neurotransmitter synthesis and sustenance of the myelin sheath. This finding is said to be correlated with clinical data of MTX-treated ALL patients with a decrease in CSF folate content (Vezmar et al. 2009). Similar results were also reported by another study where female C57B16/J mice on acute MTX (20 mg/kg, *i.p*) injection showed folate depletion concurred with increased CSF tau and reduced hippocampal cell proliferation (Elens et al. 2019).

#### Cisplatin

Cisplatin ([cis-diamminedichloridoplatinum-II] CDDP) is a platinum-based, broad-spectrum chemotherapeutic drug used in different types of malignancies (head, neck, testicular, gynecologic, non-small cell lung, and colon cancer). The cytotoxic action of CDDP is through its interaction with N7 reactive center on purine residue of DNA to form DNA adducts by intra-strand cross-linkage and activate different downstream signaling pathways (ATR [ataxia telangiectasia and Rad3-related], p53, p73, MAPK [mitogen-activated protein kinase]) thereby blocking cell division and culminating in apoptosis (Siddik 2003).

CDDP crosses BBB via copper uptake protein copper transporter (CTR1), which is expressed in the endothelial cells of BBB (Kilari 2016) and reported to achieve high concentration in the patient’s brain (Nakagawa et al. 1996). Cognitive functioning evaluated by neuropsychological tests and brain imaging studies has reported that head, neck (Gan et al. 2011), and testicular cancer (Schagen et al. 2008) patients receiving CDDP as a part of their chemotherapy regimen showed cognitive impairments. Preclinical studies have reported that CDDP crosses BBB and impairs neurogenesis in the brain (Seigers et al. 2013). A decrease in the number of dendritic spines and arborizations with the increase in the adhesion of white matter structures and cognitive impairment was proved in mice treated with CDDP (Zhou et al. 2016). Different animal studies have reported that CDDP induces cognitive impairment causing biochemical and behavioral alterations (Table 5).

**Table 5:** Different animal models of cisplatin inducing cognitive dysfunction

Species	Dose & route of administration	Comments	Reference
Male C57BL/6 J mice	2.3 mg/kg, *i.p* for 5 days injection-5 days no injection-5 days injection	• Loss of dendritic arborization and lateral fibers in cingulate cortex • Neuronal mitochondrial damage	(Chiu et al. 2016)
Male C57BL/6 J mice (5–6 months old)	2.3 mg/kg, *i.p* for 5 days injection-5 days no injection-5 days injection	• Attention deficits in 5-choice serial reaction time task • Reduced expression of synaptophysin and VGlut2	(Huo et al. 2018)
Male Wistar rats (5–6 weeks old)	5 mg/kg/week, *i.p* for 7 weeks	• Increased levels of MDA, nitrites • Reduced levels of SOD, catalase, reduced glutathione • Impaired spatial and recognition memory in MWM and NORT respectively	(Jangra et al. 2016)
Male Sprague–Dawley rats (200–250 g)	5 mg/kg/week, *i.p* for 4 weeks	• Reduced freezing in fear conditioning task • Episodic memory impairment in NORT	(Lomeli et al. 2017)
Male Wistar rats (18 months old)	5 mg/kg/week, *i.p* for 5 weeks	• Increased MDA, protein carbonyls, iNOS, and nitrites • Decreased levels of catalase, SOD and reduced glutathione	(Chtourou et al. 2015)
Male Sprague–Dawley rats (post-natal day 25)	2 mg/kg, *i.p* for 5 days	• Hippocampal-dependent impairment in NORT and fear conditioning task	(John et al. 2017)

Mitochondrial damage results in oxidative stress, inflammation, and proapoptotic signaling. C57BL/6 J mice treated with CDDP (2.3 mg/kg, *i.p*) for 5 days injection-5 days no injection-5 days injection showed loss of arborization and lateral fibers in the cingulate cortex. CDDP increased the translocation of p53 to mitochondria suggesting neuronal mitochondrial damage (Chiu et al. 2016). Another study using the same strain of mice and under the same dose of CDDP showed attention deficits manifested by the reduction in the percentage of correct responses with an increase in omissions assessed by a 5-choice serial reaction time task. CDDP reduced expression of presynaptic marker synaptophysin and glutamatergic presynaptic marker VGlut2 and negatively affecting synaptic integrity resulting in long-lasting attention deficits (Huo et al. 2018). Reports documented that a low dose of CDDP (0.1 µM) reduced dendritic spine density and induced apoptosis in the cultures of hippocampal neurons isolated from Sprague–Dawley rats (Andres et al. 2014).

CDDP (5 mg/kg/week, *i.p*) administration for 7 weeks in male Wistar rats depicted increased levels of MDA and nitrites, and reduced levels of SOD (superoxide dismutase), and reduced glutathione and catalase in the hippocampus. CDDP treatment increased the levels of different pro-inflammatory cytokines like IL-1β and TNF-α and reduced BDNF levels. It has also enhanced hippocampal NF-κB and reduced expressions of Nrf-2 (nuclear factor erythroid 2-related factor-2) and HO1 (heme oxygenase-1). Behavioral studies showed that CDDP-treated rats spent less time in the target quadrant than the control group in MWM for spatial memory assessment and showed low preference for the novel object in NORT to assess the recognition memory (Jangra et al. 2016). A similar study done in aged Wistar rats treated with CDDP (5 mg/kg/week, i.p) for 5 consecutive weeks reported increased MDA, protein carbonyls, and nitrites. It decreased the levels of catalase and SOD, and reduced glutathione with increased mRNA levels of AChE (acetylcholinesterase) and iNOS in the hippocampus (Chtourou et al. 2015).

Chronic administration of CDDP (5 mg/kg/week, *i.p*) for 4 weeks in male Sprague–Dawley rats showed less time in freezing in context fear conditioning memory task as well as no preference for either novel or familiar objects in NORT compared with the control group (Lomeli et al. 2017). Male SPF C57BL/6 mice treated with CDDP (2.3 mg/kg, *i.p*) for 5 days injection-5 days no injection-5 days injection inhibited Akt-mTOR phosphorylation through increased ATF4 (activating transcription factor 4) levels and endoplasmic stress, thereby inducing apoptosis. Cognitive function was assessed using NORT and MWM, where CDDP-treated mice showed impaired episodic and spatial memory, respectively. This was evidenced by the reduced preference for the novel object and increased escape latency compared to the control group (Yi et al. 2020). Systemic CDDP (2 mg/kg, *i.p*) administration for 5 days in Sprague–Dawley rats caused hippocampal-dependent impairment in adult rats (65 days after postnatal day) assessed using NORT and fear conditioning memory task (John et al. 2017).

#### Oxaliplatin

Oxaliplatin (OX) is an analog of CDDP with a 1,2-diamino cyclohexane carrier ligand commonly used in adjuvant and metastatic cancers (Alcindor and Beauger 2011). Like other platinum-based chemotherapeutic drugs, OX disrupts DNA replication and transcription and exerts its cytotoxic effects by forming intra-strand DNA adducts. The apoptotic effect of OX is caused by triggering different immunologic reactions, but the underlying molecular mechanisms have not been adequately elucidated (Arango et al. 2004). Administration of OX caused changes in the light junction proteins (zonula occludens-1 and F-actin) and thereby alterations in BBB (Branca et al. 2018). It has also been reported that OX-DNA adduct is more effective in exerting cytotoxic effect than adducts formed from CDDP and has a different spectrum of activity with various molecular targets and mechanisms of resistance (Raymond et al. 1998). Different preclinical studies have documented the actions of OX in inducing cognitive impairment causing dysregulation of cytokine and neurotransmitter levels, and consequent behavioral alterations (Table 6).

**Table 6:** Different animal models of oxaliplatin inducing cognitive dysfunction

Species	Dose & route of administration	Comments	Reference
Male hooded Wistar rats	Single injection 0.6,2,6 mg/kg, *i.p*	• Impairment in recognition memory in NORT • Impaired spatial memory in OLT	(Fardell et al. 2015)
Male Sprague–Dawley rats (92 days old)	Single injection 12 mg/kg, *i.p*	• Impaired renewal of fear in fear conditioning memory task	(Sharpe et al. 2012)
Male Wistar rats (12–15 weeks old)	2.4 mg/kg, *i.p* for 5 consecutive days/week for 2 weeks	• Loss of hippocampal volume	(Sadeghinezhad and Amrein 2020)
Male Sprague–Dawley rats (220–250 g)	4 mg/kg/day, *i.p* for 5 days	• Decreased recognition index in NORT • Activation TNF-α/NF-κB signaling in the hippocampus	(Zhou et al. 2020)
Male Wistar rats (150–200 g)	4 mg/kg, *i.p* for 4 times a week	• Increased MDA, protein carbonyls and glutathione depletion in brain mitochondria • Activation of caspase 3 and inactivation of Bcl-2 • Neurotoxicity and mitochondrial dysfunction	(Waseem et al. 2016)

The long-lasting impact of OX (0.6,2,6 mg/kg, *i.p*) on cognitive function was assessed in the male hooded Wistar rats at 11 months after treatment reported impairment in recognition memory rats showed by no novel object or location preference in NORT and OLT, respectively (Fardell et al. 2015). Single intraperitoneal injection of OX (12 mg/kg) in male Sprague–Dawley rats impaired the renewal of fear to an extinguished conditional stimulus in the fear conditioning memory task (Sharpe et al. 2012). Furthermore, OX (2.4 mg/kg, *i.p*) administration in male Wistar rats for 5 consecutive days/week for 2 weeks induced the loss of hippocampal volume estimated by stereology in Cavalieri’s method (Sadeghinezhad and Amrein 2020).

Short-term memory deficits were reported by the intraperitoneal injections of OX (4 mg/kg/day) for 5 days in male Sprague–Dawley rats accompanied by synaptic dysfunction. This was evidenced by decreased recognition index by OX-treated rats in NORT and showed a deficiency in Mg^2+^, which lead to the activation of TNF-α/NF-κB signaling in the hippocampus (Zhou et al. 2020).

Male Wistar rats treated with OX (4 mg/kg, *i.p*) 4 times a week showed a rise in MDA and protein carbonyl levels and significant glutathione depletion in the brain mitochondria. In addition, OX treatment caused activation of caspase 3 and inactivation of Bcl-2 with a higher concentration of cytosolic cytochrome c and lower mitochondrial cytochrome c concentration showing neurotoxicity and mitochondrial dysfunction in rats (Waseem et al. 2016).

#### Docetaxel

Docetaxel (DTX) is a second-generation, semi-synthetic taxane, an antimitotic, chemotherapeutic agent, with multiple effector targets and is widely employed for treating breast, head, neck, prostate, ovarian, and non-small cell lung cancers (Herbst and Khuri 2003; Ross et al. 2012). DTX mainly exerts its cytotoxic action by inhibiting microtubule depolymerization and amelioration of Bcl-2 and Bcl-XL gene expression. It causes cell cycle arrest at the G2/M phase providing Bcl-2 triggering series of events and apoptosis (Pienta 2001). Microtubules are the critical components of spine morphology, synaptic growth, and synaptic plasticity. So, any alterations in the microtubule network lead to cognitive deficits (Jaworski et al. 2009). Taxanes are reported to be brain penetrants and have particular toxicity in the peripheral nervous system (200 ng/g) (Roglio et al. 2009). A study on cognitive performance in older people with early-stage breast cancer reported that the likelihood of objective cognitive decline increases in patients treated with DTX after adjuvant therapy (Lange et al. 2016). Different animal studies have reported that DTX induces cognitive impairment affecting neuronal functions (Table 7).

**Table 7:** Different animal models of docetaxel inducing cognitive dysfunction

Species	Dose & route of administration	Comments	Reference
Male CD1 mice (27–33 g)	8 mg/kg, *i.p* weekly on days 0,9,18, and 28 & single injection: 8 mg/kg for 4 weeks	• Impairment in recognition memory in NORT • Impaired spatial memory in MWM	(Fardell et al. 2013a)
Male Sprague–Dawley rats (220–250 g)	Single injection 30 mg/kg, *i.p*	• Increased levels of MDA, p38α, MAPK, TNF-α, GFAP, NF-κB • Decreased levels of glutathione, SOD, catalase, Nrf-2, NCAM, and HO1	(Yardım et al. 2020)
Male Han Wistar rats (4–5 months old)	1 mg/kg/week, *i.v* for 4 weeks	• Reduced exploration and spatial memory deficits in object exploration task	(Callaghan and O’Mara 2015)
Male Hooded Wistar rats	Long term treatment (6,10 mg/kg/week, *i.p*) for 3 weeks Short-term treatment single injection (10 mg/kg, *i.p*)	• Cognitive impairments in NORT and MWM	(Fardell et al. 2013b)
Male C57BL/6 J mice (11 weeks old)	Single injection 33 mg/kg, *i.p*	• Cognitive impairments in OLT and SCRTT • Short-term memory and attentional deficits	(Seigers et al. 2014)

Intermittent administration of DTX (8 mg/kg, *i.p*) weekly on days 0, 9, 18, and 28 and single intraperitoneal injection of DTX delivery system, which continuously released 8 mg/kg for 4 weeks, in male CD1 mice exhibited significant cognitive impairment in NORT. In MWM, DTX-treated rats reported a reduction in the time spent in the target quadrant but not significantly compared with the control group (Fardell et al. 2013a). A single dose of DTX (30 mg/kg, *i.p*) in male Sprague–Dawley rats increased the levels of MDA, p38α, MAPK, TNF-α, GFAP, and NF-κB and decreased the levels of glutathione, SOD, catalase, Nrf-2, NCAM (neural cell adhesion molecule), and HO1 (Yardım et al. 2020).

Long-term spatial memory deficits were induced in male Han Wistar rats treated with DTX (1 mg/kg/week, *i.v*) for 4 weeks and assessed in object exploration task. DTX treatment reduced exploration of the removed object compared to the control animals (Callaghan and O’Mara 2015). Long-term treatment of DTX (6,10 mg/kg/week, *i.p*) for 3 weeks and short-term treatment as a single injection (10 mg/kg, *i.p*) in male hooded Wistar rats assessed cognitive impairments in behavioral paradigms like NORT and MWM. Researchers have found that DTX treatment showed impaired cognitive performance in both tasks, but no effect was observed in MWM after acute treatment (Fardell et al. 2013b). A single injection of DTX (33 mg/kg, *i.p*) in C57BL/6 J mice showed impairments in OLT and simple chronic reaction time task (SCRTT), affecting hippocampal short-term memory and attentional performance, respectively (Seigers et al. 2014).

#### Paclitaxel

Paclitaxel (PTX) is another taxane-based chemotherapeutic agent used in breast, ovary, and lung cancers. Cytotoxic action of PTX is by reorganizing the microtubule cytoskeleton and thereby blocking the G2/M phase of the cell cycle, failing mitotic apparatus formation (Horwitz 1994). Positron emission tomography detected radiolabeled PTX after intravenous administration in brain tissues implying that a small amount of drug can cross BBB (Gangloff et al. 2005). PTX-induced chemobrain animal models have reported that PTX induces cytokine deregulation and behavioral alternations (Table 8).

**Table 8:** Different animal models of paclitaxel inducing cognitive dysfunction

Species	Dose & route of administration	Comments	Reference
Male Sprague–Dawley rats (6–8 weeks old)	2 mg/kg/day, *i.p* for four days	• Neuronal apoptosis • Impaired spatial memory in MWM • Increased number of TUNEL-positive cells, TNF-α and IL-β in the hippocampus	(Li et al. 2018)
Male C57BL/6 mice (9 weeks old)	12 injections 20 mg/kg, *i.p* for 4 weeks	• Impaired spatial memory in MWM	(Huehnchen et al. 2017)
Male C57BL/6 mice	Acute: 10 mg/kg/day, *i.p* for 7 days Chronic:10 mg/kg/day, *i.p*) for 30 days	• Decrease in the testicular zinc levels and ZnT3 expression in hippocampus • Spatial memory deficits in MWM	(Lee et al. 2017)
Male Sprague–Dawley rats (10 months old)	2 mg/kg, *i.p* for 4 alternating days	• Reduced number of Ki67 and BrdU positive cells • Reduced hippocampal neurogenesis	(Panoz-Brown et al. 2017)

Male Sprague–Dawley rats after PTX (2 mg/kg/day, *i.p*) treatment for 4 days showed impaired spatial memory function with increased duration of escape latency compared with vehicle in MWM test. PTX-induced neuronal apoptosis in the hippocampus was evident through the increased number of TUNEL-positive cells. The study also reported an increase in the expression of proinflammatory cytokines TNF-α and IL-β in the hippocampus (Li et al. 2018). Reports suggested that 12 injections of PTX(20 mg/kg, *i.p*) for 4 weeks impaired spatial memory in the MWM task where the animals showed difficulty in finding the location of the escape platform during the acquisition trial and also spent less time in the target quadrant in the probe trial (Huehnchen et al. 2017).

Zinc is an important transition metal having a wide variety of biological actions. It acts as a co-transmitter at the synapses and is involved in synaptic plasticity, neurogenesis (Hirzel et al. 2006; Qian and Noebels 2005) and also necessary for the formation of postsynaptic density proteins (PSD) (Baron et al. 2006). Different types of insults to the brain have been found to alter the zinc levels in the hippocampus (Choi et al. 2014). Acute PTX (10 mg/kg/day, *i.p*) for 7 days and chronic (10 mg/kg/day, *i.p*) for 30 days in male C57BL/6 mice showed decreased testicular zinc levels and reduced expression of ZnT3 in the mossy fibers of the hippocampus. PTX treatment also induced spatial memory deficits in the MWM. This cognitive dysfunction was reversed by supplementing zinc with ZnCl_2_, increasing zinc levels and neuroblast production (Lee et al. 2017).

The molecular and cellular role of PTX-induced dyscognition was established by administering four intraperitoneal injections of PTX into mice. The researchers found decreased spine density and dendritic arborizations in the cortex and hippocampus through PKC signaling upregulation (Nguyen et al. 2020). Male Sprague–Dawley rats treated with PTX (2 mg/kg, *i.p*) on 4 alternating days reduced the number of Ki67- and BrdU-positive cells in the subgranular zone, indicating hippocampal neurogenesis (Panoz-Brown et al. 2017).

#### Temozolomide

Temozolomide (TMZ) is an alkylating agent and an FDA-approved drug for treating glioblastoma multiforme. It exerts cytotoxicity by converting intracellularly to MTIC (monomethyl triazene 5,3-methyl triazen-1-yl-imidazole-4-carboxamide). This potent methylating agent introduces methyl groups to DNA guanine bases and makes the cellular repair mechanisms unable to correct the methylated bases, followed by the formation of DNA nicks and finally apoptosis (Wesolowski et al. 2010; Zhang et al. 2012). TMZ can cross BBB and introduce direct toxicity in the brain cells causing impaired hippocampal neurogenesis (Bird et al. 2016). Preclinical studies involving TMZ have reported that it causes cognitive impairment making alterations in the biochemical and behavioral parameters (Table 9).

**Table 9:** Different animal models of Temozolomide inducing cognitive dysfunction

Species	Dose & route of administration	Comments	Reference
Male Wistar rats (12 weeks old)	18 mg/kg, *i.v* once in 5 days over 32 days	• Increased MDA and reduced SOD, catalase • Impaired episodic memory in NORT	(Pathak et al. 2020)
Male Sprague–Dawley rats (60–75 days old)	25 mg/kg, *i.p* in a cyclic manner for several weeks	• Impaired hippocampal neurogenesis and endogenous theta activity • Difficulty in learning and memory	(Nokia et al. 2012)

Male Wistar rats treated with TMZ (18 mg/kg, *i.v*) once in 5 days over 32 days showed no preference to the novel object in NORT. The antioxidant analysis showed that TMZ increased MDA and reduced SOD and catalase levels compared with the control group animals (Pathak et al. 2020).

Chronic administration of TMZ (25 mg/kg, *i.p*) in a cyclic manner for several weeks in male Sprague–Dawley rats impaired hippocampal neurogenesis and endogenous theta activity, which affected learning and memory. Along with this, researchers reported that rats treated with TMZ found difficulty in learning the association between two stimuli if a time gap separates them. Nevertheless, behaviors learned before exposure to TMZ were intact, implying that deficits are more associated with real-time learning aspects than memory (Nokia et al. 2012).

### Miscellaneous agents

Apart from commonly used agents in cancer therapy, various other drugs effectively treat different types of cancers in animal models. They have also been detected to affect cognitive function adversely. So, there is a possibility of cognitive dysfunction by using those agents, which is often unaddressed.

#### Colchicine

Colchicine is a microtubule destabilizing agent and suppresses mitosis by inhibiting cell division. Anti-tumor and anti-proliferative effects of colchicine are exerted by increasing cellular free tubulin levels and interfering in mitochondrial metabolism of cancer cells through the voltage-dependent ion channels leading to apoptotic cell death (Kumar et al. 2016; Maldonado et al. 2010). Daily or weekly therapy of low-dose intravenous colchicine was effective in 14 patients with refractory chronic lymphocytic leukemia (Weick et al. 1983). Colchicine has shown antiproliferative effects in human gastric cancer cell lines like AGS and NCI-N87 (6 ng/mL), evidenced by the upregulation of different genes, especially the DUSP1 gene leading to the suppression of extracellular signal-regulated kinase and S-phase associated protein two kinase resulting in apoptosis. The same study in nude mice (0.07 mg/kg/day) for 14 days also lowered tumor growth rates and volume ratios (Lin et al. 2016). Colchicine exhibited the anti-cancer effects in prostate adenocarcinoma cell line MAT-LyLu (MLL) and human prostate cancer cells (PC-3) and also in male Copenhagen rats treated intraperitoneally (given as 0.1% from 10 mg/ml of stock solution) for 9 days (Fakih et al. 1995). Reactive oxygen species-mediated cell death and anti-tumor effects of colchicine were also reported in human hypopharyngeal cancer cell lines (Cho et al. 2017), lung cancer cell lines (Bhattacharya et al. 2016), pancreatic cancer cell lines (Larocque et al. 2014), and breast cancer cell lines (Sun et al. 2016). But the pharmaceutical importance of colchicine for its anti-cancer activity is questionable because of its very narrow therapeutic index, so the monitoring of drug plasma concentration is necessary.

Despite its anti-cancer effects, colchicine is a neurotoxic agent that induces neurofibrillary degeneration, depletion of antioxidant defense system, and loss of cholinergic neurons with marked destruction of hippocampal granule cells and septohippocampal pathway affecting learning and memory (Table 10). Intracerebroventricular administration of colchicine is recognized as a reliable model for inducing features similar to sporadic dementia of Alzheimer’s type as in humans. It causes a subtle and harmful onset with time-dependent changes in behavioral and biochemical patterns (Neha et al. 2014).

**Table 10:** Different animal models of colchicine inducing cognitive dysfunction

Species	Dose & route of administration	Comments	Reference
Male Wistar rats (2–3 months old)	15 µg/5 µl in ACSF, *i.c.v*	• Impaired spatial memory in MWM • Increased MDA & Acetylcholine esterase (AChE) levels • Decreased catalase & SOD levels	(John et al. 2020)
Male Swiss albino mice (25–30 g)	1 & 3 µg/10 µl in ACSF, *i.c.v*	• Impaired spatial memory in MWM • Increased levels of MDA and nitrite • Decreased glutathione level	(Awasthi et al. 2012)
Male Wistar albino rats (180–200 g)	7.5 µg/5 µl in ACSF, *i.c.v*	• Increased TBARS, nitric oxide, IL-1β, IL-6, and TNF-α levels • Reduced levels of SOD and glutathione	(Essawy et al. 2019)
Male Wistar rats (80–100 g)	15,30,60,120 µg/kg in saline, *i.p*	• Deficits in food rewarding operant responding paradigm	(Bensimon and Chermat 1991)

#### Streptozotocin

Streptozotocin (STZ) is an FDA-approved treatment to manage metastatic insulinomas in humans. It exerts diabetogenic action in animals to which humans are resistant. In addition, it has nitrosourea-like cytotoxic activity causing DNA alkylation and inhibition of DNA synthesis. But it is not an effective myelosuppressive agent for human insulinomas that do not express the GLUT2 transporters (Elsner et al. 2003; Gustafson and Page 2012).

STZ is commonly used to induce cognitive deficits in animal models by exerting hyperglycemia resulting in oxidative stress and inflammatory responses, which are the significant risk factors for Alzheimer’s disease (Table 11). Morphological and biochemical alterations occur in the hippocampus due to increased MDA levels and decreased SOD levels with the dysregulated generation of TNF-α, IL-6, and IL-1β (Liu et al. 2016).

**Table 11:** Different animal models of streptozotocin inducing cognitive dysfunction

Species	Dose & route of administration	Comments	Reference
Male Wistar rats (210–230 g)	65 mg/kg, *i.p*	• Impaired spatial memory in MWM • Increased IL-1β, IL-6, and TNF-α levels	(Liu et al. 2016)
Male C57BL/6 J mice (8–10 weeks old)	180 mg/kg, *i.p*	• Impaired spatial memory in MWM • Enhanced levels of MDA, IL-1β, IL-6, and TNF-α levels • Decreased SOD level	(Wang et al. 2018)
Male Wistar rats (5–7 month old)	3 mg/kg in 10 µl ACSF, *i.c.v*	• Increased AChE, MDA, nitrite, and lactate dehydrogenase activity • Decreased level of glutathione	(Deshmukh et al. 2009)
Male albino mice (25–30 g)	3 mg/kg in 10 µl ACSF, *i.c.v*	• Impaired spatial memory in MWM and episodic memory in NORT • Enhanced levels of MDA, IL-6, and TNF-α levels	(El Sayed and Ghoneum 2020)

### Combination therapy-based chemobrain models

The primary purpose of using a combination of chemotherapeutic agents in cancer therapy is that the drugs that work by distinct mechanisms reduce the chances of developing resistance in cancer cells. Each drug can be used at the optimum dose with tolerable side effects. They may synergistically target key pathways and are helpful for patients with advanced forms of cancer who are not suitable for radiation therapy/surgery. Simultaneously, combination therapy can also lead to unwanted side effects and make it challenging to identify the responsible agents (Mokhtari et al. 2017).

Studies performed in breast cancer patients (Cheung et al. 2015), ovarian cancer patients (Correa et al. 2017), and children diagnosed with acute lymphoblastic leukemia (Ashford et al. 2010) have shown that a combination of chemotherapy regimens is associated with cognitive impairment. Combination therapies contributing to chemobrain are clinically relevant because most cancer treatments involve two or more chemotherapeutic agents. Preclinical assessment of individual agents or combination therapy and its consequent determination of the mechanism of action play a crucial role in chemobrain research because in a clinical setting, ascertaining the individual toxicity of each drug or the synergistic effects of agents in a combination regimen is often a difficult task (Matsos and Johnston 2019). Some commonly used combination therapies have been reported to cause cognitive impairments in different preclinical studies (Table 12).

**Table 12:** Combination of different chemotherapeutic agents used in different animal models and their respective changes leading to chemobrain

Species	Drugs	Comments	Reference
Ovariectomized Sprague–Dawley rats (8 weeks old)	CPP (40 mg/kg/week, *i.p*) + DOX (4 mg/kg/week, *i.p*) for 3 weeks	• Impaired contextual fear memory in classical fear conditioning task	(MacLeod et al. 2007)
C57BI6/J female mice (6 months old)	CPP (60 mg/kg/week, *i.p*) + MTX (4 mg/kg/week, *i.p*) + 5-FU (60 mg/kg/week, *i.p*) for 4 weeks	• Decrease in number of mushroom spines of dentate gyrus • Impairment in long-term memory	(Anderson et al. 2020)
Female Wistar rats (4 months old)	CPP (40 mg/kg/week, *i.p*) + MTX (37.5 mg/kg/week, *i.p*) + 5-FU (75 mg/kg/week, *i.p*) for 4 weeks	• Decreased hippocampal cell proliferation • Impaired learning and memory in MWM	(Briones and Woods 2011)
Female C57B/BL6J mice (18–20 g)	DTX (10 mg/kg, *i.p*) + DOX (10 mg/kg, *i.p*) + CPP (40 mg/kg, *i.p*) 2-day interval within 1 week	• Increased escape latency • Increased time for first entry into targeted quadrant • Increased levels of IL-6 and TNF-α & decreased levels of IL-10 and IL-4	(Shi et al. 2018)
Female Sprague–Dawley rats (10 months old)	Adriamycin (2.5 mg/kg, *i.p.*) + Cytoxan (25 mg/kg, *i.p.*) 4 doses at weekly intervals	• Impaired memory in passive avoidance test	(Konat et al. 2008)
Female BALB/C mice (3 months old)	MTX (50 mg/kg/week, *i.p.*) + 5-FU (75 mg/kg/week, *i.p.*) for 4 weeks	• Impaired cognitive performance in spatial memory, cued memory, non-matching to sample (NMTS), delayed NMTS	(Winocur et al. 2011)
Female Wistar rats (2–4 months old)	CPP (40 mg/kg/week, *i.p*) + MTX (37.5 mg/kg/week, *i.p*) + 5-FU (75 mg/kg/week, *i.p*) for 2 weeks	• Impaired working memory in OLT	(Larkov et al. 2016)
Female C57B/BL6J mice (16 weeks old)	CPP (50 mg/kg/week, *i.p*) + DOX (2 mg/kg/week, *i.p*) for 4 weeks + PTX (2 mg/kg/week, *i.p*) for 4 weeks	• Impaired spatial memory • Decreased dendrite length in the hippocampus	(McElroy et al. 2020)
Female BALB/C mice (8 weeks old)	CPP (25 mg/kg/week, *i.v*) + DOX (2.5 mg/kg/week, *i.v*) for 4 weeks	• Impaired spatial memory in MWM	(Philpot et al. 2019)
Male hooded Wistar rats (265–369 g)	Single injection OX (12 mg/kg, *i.p*) + 5-FU (75 mg/kg, *i.p*)	• Impaired contextual fear recall • Impaired episodic memory in NORT	(Fardell et al. 2012)
Male Swiss albino mice(8–10 weeks old)	CPP (50 mg/kg/week, *i.p*) + MTX (5 mg/kg/week, *i.p*) + 5-FU (5 mg/kg/week, *i.p*) for 3 weeks	• Impaired spatial memory in MWM • Increased MDA and decreased catalase	(Kinra et al. 2020)
Male C57BL/6Hsd mice (7–8 weeks old)	MTX (37.5 mg/kg/week, *i.p*) + 5-FU (75 mg/kg/week, *i.p*) for 4 weeks	• Decreased ability to gate incoming auditory stimuli • Decreased adaptation to novel objects in NORT	(Gandal et al. 2008)
Female Sprague–Dawley rats (12 months old)	CPP (40 mg/kg/week, *i.p*) + MTX (37.5 mg/kg/week, *i.p*) + 5-FU (75 mg/kg/week, *i.p*) for 4 weeks	• Increased levels of inflammatory mediators like TNF-α, IL-1β, and COX-II decreased level of anti-inflammatory mediator IL-10 • Decrease in the level of oligodendrocyte precursor cells (OPCs) and reduction in myelin sheath thickness and myelinated axons	(Briones and Woods 2014)
Male Swiss Webster mice (20–25 g)	MTX (32 mg/kg/week, *i.p*) + 5-FU (75 mg/kg/week, *i.p*) for 3 weeks	• Impaired learning and memory in autoshaping responding procedure	(Walker et al. 2011)

### Cyclophosphamide + methotrexate + 5-fluorouracil (CMF)

CMF regimen is a common chemotherapy regimen used in breast cancer patients (Koppelmans et al. 2012). C57BI6/J mice, when administered with CPP (60 mg/kg/week, *i.p*), MTX (4 mg/kg/week, *i.p*), and 5-FU (60 mg/kg/week, *i.p*) for 4 weeks, reported a decrease in the number of mushroom spines in the dentate gyrus. Impairment in long-term memory was also observed in Y-maze and MWM tasks (Anderson et al. 2020). It was also reported that female Wistar rats when treated with CPP (40 mg/kg/week, *i.p*), MTX (37.5 mg/kg/week, *i.p*), and 5-FU (37.5 mg/Kg/week, *i.p*) for 4 weeks showed decreased hippocampal proliferation evidenced by a decrease in the number of BrdU-labeled cells in the dentate gyrus of the hippocampus. Spatial memory deficit was also observed in the MWM task shown by a reduction in path length (Briones and Woods 2011). Male Swiss albino mice treated with CPP (50 mg/kg/week, *i.p*), MTX (5 mg/kg/week, *i.p*), 5-FU (50 mg/kg/week, *i.p*) for 3 weeks showed impairment in spatial memory, which was evidenced by a decrease in the retention time and increase in the escape latency assessed using MWM task (Kinra et al. 2020). Female Wistar rats, when subjected to CMF treatment (40 mg/kg/week, i.p, 37.5 mg/kg/week, i.p, 75 mg/kg/week, i.p) for 2 weeks, showed lower discrimination index in novel object location task where the animals showed no preference for the object placed in the novel location(Briones and Woods 2014).

#### Cyclophosphamide + doxorubicin

Another important chemotherapeutic regimen reported to cause cognitive impairment in animals is the combination of CPP and DOX. A study involving 8-week-old ovariectomized Harlan-Sprague Dawley rats treated with CPP (40 mg/kg/week, *i.p*) and DOX (4 mg/kg/week, *i.p*) for 3 weeks exhibited decreased freezing behavior in contextual fear conditioning task, which indicates a specific deficit in hippocampal-dependent learning and memory (MacLeod et al. 2007). Similarly, ovariectomized C57BL/6 J mice when administered with DOX (2 mg/kg/week, *i.p*) and CPP (50 mg/kg/week, *i.p*) for 4 weeks showed a decreased density of stubby spines in the dentate gyrus of hippocampus exhibiting memory deficits (Kang et al. 2018). Ten-month-old female Sprague Dawley rats treated with intraperitoneal injections of four doses of Adriamycin (2.5 mg/kg/week) and cytoxan (25 mg/kg/week) showed impaired memory, which was measured by passive avoidance test. Here, a decrease in latency time (time taken to enter the dark compartment) was seen in CPP + DOX-treated group eliciting brain dysfunction (Konat et al. 2008). In another study, adult female BALB/C mice, when treated with DOX (2.5 mg/kg/week, *i.v*) followed by CPP (25 mg/kg/week, *i.v*) for 4 weeks, exhibited spatial memory deficits in the MWM task shown by a decrease in the percentage of entries to the target zone (Philpot et al. 2019). Administration of CPP (40 mg/kg/week, *i.v*) and DOX (4 mg/kg/week, *i.v*) in ovariectomized female Sprague–Dawley rats for 4 weeks showed higher levels of TNF-α, GM-CSF, VEGF, and NF-κb in the hippocampus (Bagnall-Moreau et al. 2019). Male Wistar rats treated with CPP (50 mg/kg/week, *i.p*) and DOX (2 mg/kg/week, *i.p*) for 4 weeks could not discriminate between novel and familiar spatial location in novel location recognition task (decrease in exploration time with novel location). Also, spontaneous alternation task was impaired in chemo-treated group in Y-maze test (Kitamura et al. 2017).

#### Methotrexate + 5-fluorouracil

MTX and 5-FU combination is also reported to cause dyscognition in different animal models. For example, female BALB/C mice when subjected to MTX (50 mg/kg/week, *i.p*) and 5-FU (75 mg/kg/week, *i.p*) for 4 weeks showed spatial memory deficits (spent less time in the target zone) in MWM, and there was an increased tendency to make errors assessed by DNMTS task (delayed matching to sample task) (Winocur et al. 2011). Similarly, male C57BL/6 Hsd, when treated with 4 weekly injections of MTX (37.5 mg/kg/week, i.p) and 5-FU(75 mg/kg/week, i.p), caused reduced ability to gate incoming auditory stimuli (reduced adaptation of novel object in NORT, hyperactive responsive in fear conditioning memory task) (Gandal et al. 2008). Male Swiss-Webster treated with MTX (32 mg/kg/week, *i.p*) and 5-FU(75 mg/kg/week, *i.p*) for 3 weeks produced acquisition and retention deficits in an autoshaping procedure. Here animals were trained to acquire nose-poke response synchronized with tone to receive food as the reward (Walker et al. 2011).

Apart from the abovementioned combination of chemotherapeutic agents, there are other combination regimens in cancer therapy that are found to cause cognitive impairment in different animal studies. Female C57/BL6J when treated with DTX (10 mg/kg, *i.p*), Adriamycin (10 mg/kg, *i.p*), CPP (40 mg/kg, *i.p*) at 2 days interval in 1 week reported impairment in spatial memory shown by increased escape latency and reduced frequency of entry to the target quadrant. Higher levels of TNF-α and IL-6 and decreasing levels of IL-4 and IL-10 in the hippocampus were also reported as a result of chemotherapy (Shi et al. 2018). Male hooded Wistar rats when treated with a single intraperitoneal injection of OX (12 mg/kg, *i.p*) and 5-FU (75 mg/kg, *i.p*) showed a lower preference for the novel object in NORT. The treatment also impaired contextual fear memory recall (reduced freezing behavior) in fear conditioning task (Fardell et al. 2012). Female C57BI6/J mice, when received 4 weekly intraperitoneal injections of DOX (2 mg/kg/week, i.p) and CPP (50 mg/kg/week, i.p) followed by PTX (5 mg/kg/week, i.p), showed significant spatial memory deficits in MWM task (decreased time spent in the target quadrant during retention trial) and reduced the dendritic length and complexity in the dentate gyrus and CA1 regions of the hippocampus (McElroy et al. 2020).

### Induction of chemobrain in tumor animals

Assessing the role of commonly used chemotherapeutic agents in impairing cognitive function in healthy animals gives only the basic idea of the extent of dyscognition attributed to these agents. Clinically, chemotherapy is given only when the cancer is diagnosed. So, it is necessary to consider the potential of chemotherapeutic agents to induce a cognitive decline in tumor-bearing animals. In addition, several reports suggest that cancer itself can contribute to conditions leading to cognitive impairments (Meyers et al. 2004; Schagen et al. 2014).

#### Chemically induced mammary carcinoma model

N-methyl-N-nitrosourea (NMU) is one of the commonly employed models for inducing mammary carcinoma in rodents. It directly alkylates DNA and is regarded as the simplest method to offer the characteristics of human mammary carcinoma (Tsubura et al. 2011). However, chemically induced models have some limitations, like the requirement of higher doses, non-specific target effects, and the toxicity of carcinogens, which can confer harmful impacts to the handlers (Faustino-Rocha et al. 2015).

Ramalingayya et al. reported that male Sprague–Dawley rats treated with NMU (50 mg/kg, *i.p*) followed by DOX (2.5 mg/kg, *i.p*) treatment impaired episodic and spatial memory functions in NORT and MWM. DOX treatment was started after confirming mammary carcinoma development, which took around 8–10 weeks and injected once every 5 days over 50 days. DOX-treated NMU rats showed a significant reduction in the recognition index (RI) and discriminative index (DI) in NORT, and decreased the time spent in the target quadrant with increased escape latency compared to the tumor control animals. There was also a significant decrease in the levels of antioxidant enzymes like catalase and SOD and an increase in the levels of proinflammatory cytokine TNF-α in the frontal cortex and hippocampus of NMU + DOX rats. According to the study, an overall tumor-only effect was lacking in contributing to the cognitive deficits that were found to be mainly attributed to the chemotherapy (Ramalingayya et al. 2016, 2019).

#### Cancer cell line-derived carcinoma models

Cancer cell lines have been widely used to understand cancer biology and verify different hypotheses to enhance treatment efficacy. But sometimes, they lack clinical predictive power making them sum up cancer’s diversity to a limited capacity. It is difficult to maintain transplantable cancer models in all the laboratories because of the individual specifications. The neurocognitive assessments require exposure of immunocompromised animals to the external environment for longer periods, affecting the animal’s overall health status (Gillet et al. 2013).

Seigers et al. assessed the involvement of tumor growth in hippocampal cell proliferation and discerned whether the presence of tumor reinforces the effect of the chemotherapeutic agent, MTX. Male Buffalo rats have subcutaneously injected with Morris Hepatoma 7777 cells (7.5–10 million cells/1 ml PBS) between the shoulder blades, followed by MTX (100 mg/kg, *i.p*) administration. Hippocampal cell proliferation was assessed by quantifying Ki67-positive cells where the reduction was significant in the Hepatoma + MTX group compared to the hepatoma alone group. This points out that the presence of the tumor did not amplify the harmful effects of MTX on cell proliferation in the hippocampus (Seigers et al. 2010). A similar study was reported where the role of tumor in the hippocampal dysfunction in conjunction with MTX was determined. Female C3H/HeN mice were subcutaneously injected with FM3A (1*10^7^cells/1 ml of PBS) into the right flanks to initiate tumor growth. After 1 week of inoculation, MTX (40 mg/kg, *i.p*) was administered, followed by the behavioral and immunohistochemical analysis. MTX + FM3A animals showed a significant reduction in the cross-over latency compared with the tumor alone group in the passive avoidance test. Apart from this, increased levels of microglia marker Iba1 (ionized calcium-binding adapter molecule 1) and proinflammatory markers iNOS and COX-II were found in the FM3A + MTX-treated group (Yang et al. 2012).

Female athymic rats were injected with SKOV3.ip1 cell lines intraperitoneally in the lower left quadrant and subcutaneously into the right flank, followed by five splited doses of CDDP (25 mg/kg, *i.p*) administration. In neurocognitive assessment using NORT, rats could differentiate the novel and familiar objects before the chemotherapy regimen. But post-chemotherapy impaired the ability to discriminate both objects, implying dyscognition (Pearre et al. 2020).

Himmel et al. reported the role of cognitive impairment in CPP + DOX chemotherapy in triple-negative breast cancer (TNBC) mouse xenograft model. Female athymic nude mice were subcutaneously injected with MDA-MB-231 cells (5*10^5^ cells/0.1 ml/mouse) into the right flank of the mice, followed by the administration of a single intravenous injection of DOX (5 mg/kg) + CPP (10 mg/kg). H&E staining and immunohistochemistry analysis showed decreased DCX-positive cell expression compared to tumor control, which signifies the neural plasticity impairments in the chemotherapy-treated tumor mice (Himmel et al. 2016).

Immunodeficient female mice (nu/nu athymic) were subcutaneously injected with the fresh specimen of the tumor (TumorGraft™) into the flanks, followed by the treatment with DOX/PTX/CPP. The results showed the deregulation of different miRNA families, especially miR-200 and miR-183/96/182 clusters in tumor-bearing and chemotherapy-treated animals (Kovalchuk et al. 2017). Dysregulation of miRNA leads to alteration in the levels of BDNF, which plays a vital role in cognition and memory (Baydyuk and Xu 2014).

### Induction of chemobrain in transgenic animals

Transgenic mouse models have been developed by inserting foreign DNA into the genome of the mouse. They have several advantages over xenograft models. The former allows the reproduction of genetic instability and mechanisms similar to human cancer and can represent different molecular stages of tumor transformation. But an important limitation of the transgenic model is that the identical phenotypes are exhibited due to different genotypes in mice (Conmy and Nasheuer 2010). Nevertheless, transgenic mouse models are used extensively and play an essential role in cancer research.

Different studies have already proved the role of the E4 allele of APOE (Apolipoprotein E) in increasing the risk of Alzheimer’s disease (Ward et al. 2012; Neu et al. 2017). A study conducted among long-term survivors of breast cancer and lymphoma treated with a standard dose of chemotherapy reported that survivors with at least one E4 allele showed impairment in visual memory, spatial ability, and psychomotor functioning compared to those without the E4 allele (Ahles et al. 2003). The role of APOE4 in CICI was assessed in a mouse model to understand the vulnerability of APOE4 carriers to cognitive impairment following chemotherapy. Thus, APOE-targeted replacement mice have become an important model for examining chemotherapy-induced cognitive impairment (Speidell et al. 2018). Aged (12 months) female C57BL6J APOE3 and APOE4 knock-in mice were subjected to two intraperitoneal injections of DOX (5 mg/kg) 1 week apart. Spatial learning and memory assessed using Barne’s maze showed that the total latency of APOE4 control mice was comparable to that of APOE3 control mice. DOX treatment showed no effect in APOE3 knock-in mice, whereas in APOE4 knock-in mice, the treatment led to increased total escape latency (to learn the target hole) compared to the APOE4 control mice. In the probe trial, DOX + APOE4 and APOE3 mice took a significant increase in time to enter the target hole. Anatomical brain assessment also showed a mild decrease in the grey matter volume in the frontal cortex in the APOE4 + DOX animals compared to other groups (Demby et al. 2020).

Thy1-YFP-H line transgenic mice treated with DTX (10 mg/kg, *i.p*) + DOX (10 mg/kg, *i.p*) + CPP (40 mg/kg, *i.p*) with 2 days interval and within 1 week showed a significant increase in dendritic spine elimination rate and loss of spine in the cortical neurons compared with the controls using transcranial two-photon imaging techniques. In addition, there was a substantial increase in the level of cytokines like TNF-α and 1L-6 in the frontal cortex and hippocampus (Shi et al. 2019).

FVB/N-Tg (MMTV-neu) 202 Mul/J mouse is an established transgenic model for breast cancer and is slow processing, exhibiting similarities in tumor transformation in humans. MMTV-neu oncogenic mice were treated with MTX (37.5 mg/kg/week, *i.p*) + 5-FU (50 mg/kg/week, *i.p*) for 3 weeks. Animals subjected to tumor + chemotherapy showed impairment in hippocampal-dependent spatial memory in MWM and frontal cortex-dependent cued memory in the conditional associative learning task and working memory in the NMTS learning task compared to the tumor alone group. The study reported that transgenic mice without chemotherapy also exhibited impaired learning effects but not as prominent as that of animals with tumor + chemotherapy. Chemotherapy-treated tumor-bearing mice showed cytokine deregulation explained by increased levels of IL-6, TNF-α, RANTES (regulated upon activation, normal T cell expressed and presumably secreted), and impaired neurogenesis indicated by the decreased number of DCX positive neurons (Winocur et al. 2018).

So, even though some of the studies involving tumor-bearing animals confirm that tumor may also contribute to cognitive dysfunction, it is found to be prominent in animals with chemotherapy.

### Translational aspects in chemobrain research

To date, no drug interventions are approved for preventing cognitive impairment associated with chemotherapy. But medications that are used in other neurological conditions could be employed to provide symptomatic relief. For example, donepezil (first-line drug treatment for Alzheimer’s disease) showed improvement in verbal memory aspects (Hopkins Verbal Learning Test-Revised) in female breast cancer survivors 1–5 years following chemotherapy but not in other cognitive measures (Lawrence et al.^2016^). Currently, N-acetylcysteine, an antioxidant, is under clinical trial to determine its efficacy in mitigating chemotherapy-related cognitive impairment induced by platinum therapy in ovarian cancer patients (National Library of Medicine [NLM], NCT04520139 2020). N-acetylcysteine was already found to prevent cisplatin-induced cognitive impairment in an ovarian cancer xenograft model in the rat (Lomeli 2019).

Apart from CNS, chemotherapeutic agents can affect different organ systems and induce several neurological complications. These are manifested in various ways such as neuropathy, encephalopathy, seizures, cerebellar dysfunction, headache, and vision changes (Stone and DeAngelis 2016). The extensive bidirectional communication between CNS and gastrointestinal microbiota (microbiota-gut-brain axis) has an important influence on CNS by affecting behavioral and cognitive paradigms (Subramaniam et al. 2020). Chemotherapeutic agents were reported to disrupt the protective and immunomodulatory effects offered by microbiota, causing alterations in the microbial community. These alterations were found to be direct indicators of systemic inflammation shown by microglial activation in the prefrontal cortex and hippocampus. This interaction between the gut and CNS can be utilized to explore the microbiome as a therapeutic target for CICI. Psychobiotics (microbial treatment therapy with psychological benefits) could be a relevant treatment strategy for CICI. Different preclinical studies have demonstrated the cognitive benefits offered by several orally administered microbial strains (Bajic et al. 2018; Loman et al. 2019; Ciernikova et al. 2021).

Most of the chemotherapeutic drugs are processed through the liver (Ramadori and Cameron 2010). Abnormalities like sinusoidal obstructive syndrome, steatosis, acute hepatitis, liver cirrhosis, and even liver failure were found to be associated with chemotherapy. These chronic inflammatory liver diseases are directly correlated with the changes in central neural transmission and result in psychological alterations (Sharma et al.^2014^). Peripheral inflammation and the subsequent release of different pro-inflammatory mediators (TNF-α, IL-1β, IL-6) resulting from hepatic injury led to neuroinflammation by different mechanisms. This causes structural and functional alterations in the hippocampus (Izadpanahi et al. 2020; Brown et al. 2021). Therefore, anti-inflammatory therapies that focus on liver inflammation could evolve as therapeutic interventions in CICI.

## Conclusion

Each drug used in cancer therapy is anticipated to exert a wide range of biological effects and even gets further complicated when used in combination with other drugs which is reflected in the state of chemobrain. Variability in genetic elements and treatment regimens are few among different factors contributing to the heterogeneity in the pathophysiological conditions of chemobrain. So, focusing on molecular mechanisms of each chemotherapeutic agent can be aimed to modify chemobrain through a targeted approach, which may reduce the toxicities of chemotherapeutics. Animal models covering the full spectrum of the disease are practically impossible but can be employed to test different hypotheses on each pathophysiological state. CICI in healthy animals serves as an initial phase of the study to see the extent of alterations in biochemical and behavioral states caused by individual drugs and the combination of different chemotherapy drugs. Chemobrain induction in tumor-bearing animals gives a clearer picture of the condition in the presence of cancer or after the recovery period. This further provides the evaluation of different pharmacological agents for improving cognitive function without compromising the anti-cancer potential of chemotherapeutics. So, co-administration of chemotherapeutic agents with the protective element can be evaluated extensively in preclinical studies. Because the translational approaches involving these studies should be addressed cautiously since their potential interference could adversely affect the therapeutic efficacy of chemotherapeutic agents. Identifying and determining anatomical regions and biomarkers involved in the cognitive pathway of chemobrain and developing better animal models that can manifest the complexities of disease conditions are necessary to develop new therapeutic strategies to combat CICI.
